# Assessment of the Eutrophication-Related Environmental Parameters in Two Mediterranean Lakes by Integrating Statistical Techniques and Self-Organizing Maps

**DOI:** 10.3390/ijerph15030547

**Published:** 2018-03-19

**Authors:** Ekaterini Hadjisolomou, Konstantinos Stefanidis, George Papatheodorou, Evanthia Papastergiadou

**Affiliations:** 1 Laboratory of Marine Geology and Physical Oceanography, Department of Geology, Patras University, 26504 Patras, Greece; George.Papatheodorou@upatras.gr; 2 Department of Biology, University of Patras-University Campus Rio, 26500 Patras, Greece; kstefani@chi.civil.ntua.gr (K.S.); evapap@upatras.gr (E.P.); 3 Sector of Water Resources and Environmental Engineering, School of Civil Engineering, National Technical University of Athens, 15780 Athens, Greece

**Keywords:** PCA, cluster analysis, self-organizing map, neural networks, nutrients, Mediterranean lakes

## Abstract

During the last decades, Mediterranean freshwater ecosystems, especially lakes, have been under severe pressure due to increasing eutrophication and water quality deterioration. In this article, we compared the effectiveness of different data analysis methods by assessing the contribution of environmental parameters to eutrophication processes. For this purpose, principal components analysis (PCA), cluster analysis, and a self-organizing map (SOM) were applied, using water quality data from two transboundary lakes of North Greece. SOM is considered as an advanced and powerful data analysis tool because of its ability to represent complex and nonlinear relationships among multivariate data sets. The results of PCA and cluster analysis agreed with the SOM results, although the latter provided more information because of the visualization abilities regarding the parameters’ relationships. Besides nutrients that were found to be a key factor for controlling chlorophyll-a (Chl*-*a), water temperature was related positively with algal production, while the Secchi disk depth parameter was found to be highly important and negatively related toeutrophic conditions. In general, the SOM results were more specific and allowed direct associations between the water quality variables. Our work showed that SOMs can be used effectively in limnological studies to produce robust and interpretable results, aiding scientists and managers to cope with environmental problems such as eutrophication.

## 1. Introduction

Freshwater quality has declined in the last decades throughout Europe, due to various environmental issues related to anthropogenic activities. Eutrophication is considered as one of the most important environmental problems that affects freshwater, coastal, and marine ecosystems worldwide [1]. Freshwater lakes are major providers of water for several purposes, such as water supply, drinking, irrigation, and so forth. Eutrophication has a negative impact on water quality, with ecological and socioeconomic consequences.

Eutrophication triggers various physical and chemical changes in the aquatic environment that may cause the blooming of certain harmful-toxin-producing algae (cyanophyta), which are known to create health issues for organisms living in the lakes as well as humans [2]. Because of the adverse effects of eutrophication, the necessity for mitigating eutrophic phenomena and recovering water quality has become a priority for environmental scientists [1]. The effect of eutrophication on public health is so serious that cyanobacteria have been characterized as potential key hazardous pollutants by the European Water Framework Directive (2000) (2000/60/EC) [3]. Water sports, such as swimming, in eutrophic lakes and the consumption of seafood and drinking water contaminated with cyanotoxins are the main reasons for human illness related to cyanotoxins. It is reported that about 60,000 intoxication incidents, with an overall mortality rate of 1.5%, take place per year globally because of algal toxins [4].

Artificial neural networks (ANNs) are considered to be a computational modelling tool that is widely used in solving many complex real-world problems [5,6]. In recent years, ANNs have become a desirable tool that is applied in many scientific topics [7], such as air pollution, precipitation, water quality, and classification of rainfall prediction [8]. The ability of ANNs to learn from the known data without being affected by nonlinearity and their classification potential qualify them as being superior to other traditional statistical tools [9]. Modelling studies that used both ANNs and multiple linear regression (MLR) methods found that ANNs produced better modelling results than MLR in most cases [6]. The main weakness of MLR models as compared with ANN models is that they are based on a linear relationship between input and target variables [9].

ANNs are divided into two main categories: ANNs with supervised learning and ANNs with unsupervised learning. The most popular supervised ANN is the multilayer perceptron with a backpropagation algorithm, while the most popular unsupervised ANN is the Kohonen self-organizing map (SOM) [10]. According to Peeters et al. [11], the SOM has recently started to be used in exploratory data analysis, e.g., Tsai et al. [12], Ejarque-Gonzalez & Butturini [13], and it can help with summarizing available data and extracting useful information. The SOM can deal with the phenomenon of nonlinearity, handle noisy data, and be updated easily [14]; because of this potential, the SOM algorithm is considered as a powerful tool in exploratory data analysis and the clustering of multivariate data sets [11]. The importance of the SOM for water quality management is significant, as the SOM has the potential to analyze multidimensional ecological data and simplify them into visual information that is helpful to understanding the ecological process [15].

Application of the SOM algorithm is widely used in the environmental sciences, and especially in studies examining water quality. For example, Recknagel et al. [16] used SOMs to evaluate the seasonality effect over two adjacent lakes in response to eutrophication control. A SOM was used by Oh et al. [17] to cluster the phytoplankton communities from a reservoir. In the study of Cheng et al. [18], the SOM was used to classify fish communities in shallow lakes, based on several biotic and abiotic factors such as water depth, transparency, and dissolved oxygen. The multi-relationships between fish species and river water quality parameters were examined with the use of a SOM by Tsai et al. [12]. In their study, Park et al. [10] used a SOM to classify 23 different water types, such as streams, lakes, rivers, canals, and ponds. The SOM classified the sampling sites into five clusters based on environmental parameters, and related them with species richness. In another modelling study, Tota-Maharaj & Scholz [19] applied the SOM to simulate microbial data from the effluent of pavement systems used to treat stormwater runoff, with a good accuracy, having a minimum correlation of *R* = 0.751 between the real and predicted data, and using as the model’s inputs parameters that are not expensive to measure; whereas the measurement of microbial pathogens concentrations is a time-consuming and expensive procedure. These examples demonstrate the applicability of the SOM algorithm not only in ecological research, but also in water management studies.

The objective of this study was twofold. First, we assessed the interactions of the environmental parameters related to the algal productivity of two transboundary Greek Lakes-Megali Prespa (or Great Prespa) and Mikri Prespa (or Small Prespa)—by applying a combination of a PCA, a cluster analysis, and a SOM algorithm. Second, we compared the results among the different techniques to evaluate the effectiveness of SOMs in relation to more “traditional” tools such as PCA and cluster analysis. The importance of this work also lies within the fact that there is no other published work using a SOM to study the water quality parameters related to the eutrophication of Greek lakes, to the knowledge of the authors. Therefore, this study could set the basis for the modelling with SOMs of additional Greek lakes that are impacted by the effects of eutrophication. Additionally, the results of this study can be useful for exploring the mechanisms associated with algal productivity in the studied area. 

## 2. Materials and Methods 

### 2.1. Study Area and Data Collection

**Figure 1:**
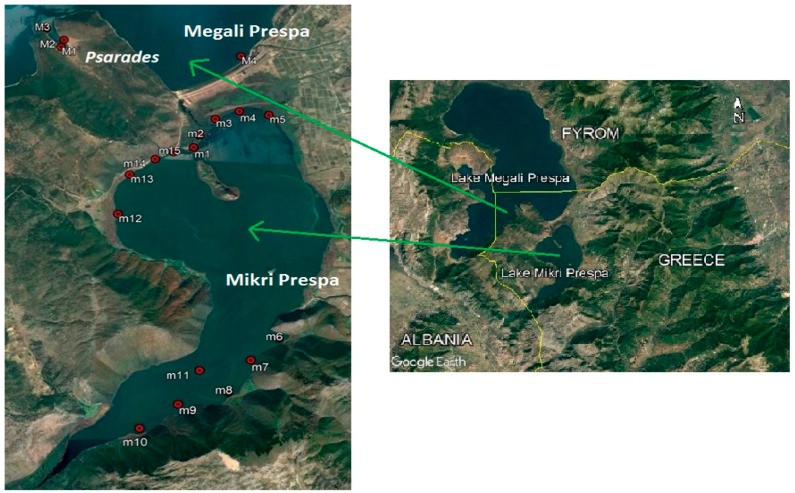
Map of the studied transboundary Lakes Megali and Mikri Prespa in northwestern Greece, with the sampling sites marked in red.

The transboundary Prespa area is a geographically remote area located in northwestern Greece. It is an area of great ecological importance, and has been declared a national park (in 1974), a Ramsar wetland of international importance (in 1987), an important bird area (in 1983), and a Natura 2000 site [20]. Lakes Megali Prespa, at 849 m above sea level (a.s.l.) [21], and Mikri Prespa, at 853.5 m a.s.l. [22], are surrounded by mountains and located in the transboundary Prespa area shared by Greece, Albania, and the Former Republic of Macedonia (FYROM) (Figure 1).

The climate of the area is characterized as sub-Mediterranean with continental influences, with frequent snowfall in the winter and summer rain drops [21]. The transboundary catchment’s area covers about 1300 km^2^ [21], and the permanent population of the Greek part is estimated at about 1500 residents [20]. Megali Prespa is a large, deep lake, covering 254 km^2^, with a 14 m mean and 48 m maximum water depth [23,24]. Mikri Prespa is a shallow lake, with approximately 48 km^2^ of surface area, and a maximum water depth of 8 m [25,26,27] and mean depth of 4.1 m [22,26,27]. The two lakes formed a single lake in the past, but nowadays are distinct and connected through an artificial channel [21]. Lake Mikri Prespa is supplied with water only seasonally through surface runoff, mainly from small rivers [22], and overflows into Lake Megali Prespa [23,28].

The inflow from Lake Mikri Prespa is about 9%, while the rest of the water inflow into Megali Prespa is from small streams (56%) and direct precipitation (35%) [29]. Lake Megali Prespa has no surface outflow, but is connected through karstic channels to the neighbouring Lake Ohrid [21].

The trophic status of Lake Mikri Prespa is characterized as eutrophic, and prolonged cyanobacterial blooms occur, which may start in spring and persist until December, favoured by the warm climate [30]. Lake Megali Prespa is considered mesotrophic [26,28], with summer bottom anoxia and an average total phosphorus concentration of 31 mg·m^−3^ [28]. 

Water samples were collected from a total of fifteen sampling sites in Lake Mikri Prespa and four sites in Lake Megali Prespa (Figure 1). Samplings were carried out on a seasonal basis during spring, summer, and autumn, for the monitoring period of 2006–2008 [27]. The measured environmental parameters were: pH, surface dissolved oxygen (DO), electrical conductivity (EC), Secchi disk (SD) depth, water depth, surface water temperature (WT), total phosphorus (TP), dissolved inorganic nitrogen (DIN), and chlorophyll-a (Chl-a).

The parameters were measured at several sampling sites within the Greek part of the lakes for each monitoring season. Most of the samples from Lake Megali Prespa were collected from the littoral zone of the lake near the Psarades village, located south of Lake Megali Prespa, and no samples from the pelagic zone were included. The statistical description of the data is given in Table 1. More details regarding the monitoring process and the measurement of environmental parameters are given by Stefanidis & Papastergiadou [27].

### 2.2. Statistical Methods and Theoretical Background 

Principal component analysis (PCA) is a multivariate data analysis method used to reveal patterns in large data sets [31]. PCA is a mathematical dimension reduction procedure, used to reduce the number of variables of a data set to a smaller number of variables, without information loss of the initial data set [32]. PCA transforms the data into a new set of variables (or coordinates), called principal components (PCs) [31]. The use of principal components instead of the initial variables is a more reliable way to represent relationships, because the system’s noise is reduced [33]. In this modelling study, the environmental parameters were normalized prior to any calculations, and all the samples that contained missing values were removed from the data set. The parameters were standardized by subtracting the sample mean from each observation and then dividing by the sample standard deviation. The simulation results for the statistical methods were carried out using the MatLab software. Only the PCs that explain at least 10% of the variance are considered for this study, as the other PCs contributed very little.

Cluster analysis is a technique that classifies objects into different groups based on their characteristics [34]. The aim of cluster analysis is to find groups (clusters) with homogeneous properties out of heterogeneous large samples, with high internal homogeneity within clusters and high heterogeneity between clusters [31]. High levels of similarity among objects are indicated by a small value in a distance matrix and large values in proximity or similarity matrices [35]. Cluster methods are divided into four categories: hierarchical methods, partitioning methods, overlapping cluster procedures, and ordination techniques [31].

Euclidean distance and Ward agglomerative methods were used for the cluster analysis. The clusters number can be calculated with the use of a cutoff line for the dendrogram, based on the Sneath’s index of cluster significance [36]. The less restrictive significance criterion of Sneath’s index (2/3 of *Dmax*) and the strict significance criterion of Sneath’s index (1/3 of *Dmax*) may be used, where *Dmax* is the maximum of the distance measure *D*.

### 2.3. Self Organizing Map Theory

**Figure 2:**
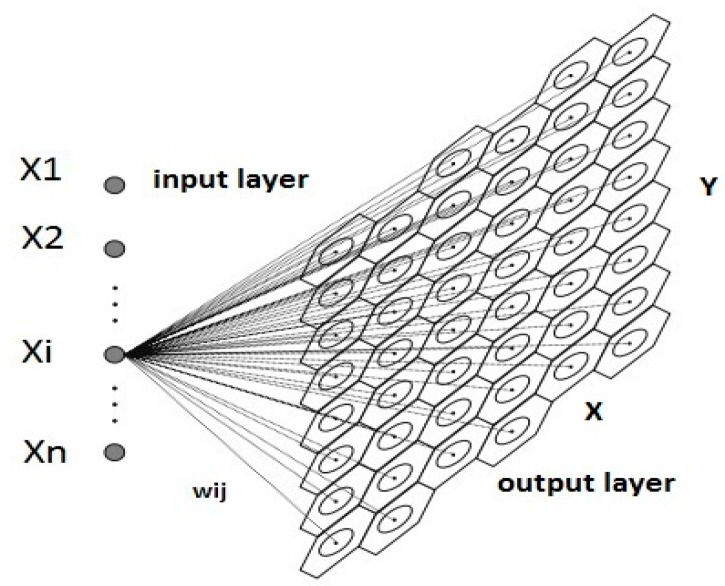
Projection of n-dimensional data into a two-dimensional (*XY*) space (output layer) with the use of a SOM; where *X1*, *X2*,*…*,*Xn* are the input variables, *n* is the input variable’s number, and *w_ij_* is the synaptic weight that is connecting the *i* input variable with the *j* node.

The Kohonen SOM is an unsupervised ANN. The SOM has the ability to learn without being given the associated output values for the corresponding input data, and the desired output is not known a priori. The goal of the learning process is to classify the input data according to their similarity [11]. SOMs are a very practical tool for data visualization; also, SOMs can be used for prediction and correlation analysis, mostly with visual representation [37]. One of the various applications of the SOM algorithm is the finding of statistically significant dependencies among the variables in a multidimensional data sample, where two highly correlated variables produce two similar component planes [38]. A SOM projects high-dimensional data into a low-dimension space [39]. This is usually a two-dimensional space, so the neurons are arranged in two dimensions (see Figure 2), because the visual summary of the output is more understandable [40]. The sample data can be clustered either as manually determined by a U-matrix or can be automated by a clustering algorithm implemented in the SOM, and usually the hierarchical clustering algorithm is applied [41,42].

The SOM is consisted of an input layer and an output layer that are connected with computational weights [43,44]. The output layer consists of neurons that are arranged in a hexagonal or rectangular grid and are fully interconnected [11]. The input patterns, usually after normalization, are imported through the neurons in the input layer. The SOM algorithm can be summarized into the following steps [42]:

1. Weight vector initialization with random values.
2. Use of a distance measure, usually the Euclidean distance, to find the best-matching unit (BMU).
3. Movement closer to the input vector by updating the weight vector of the BMU and the neighboring neurons.

The Euclidean distance (*D_i_*) mentioned above is described by the following equation, and calculates the distance measure between the input vector and the *i* weight vector [44,45]:

(1) $$D_{i}=\sqrt{\sum_{j=1}^{R}{\left(p_{ij}-w_{ij}\;\right)}^{2}}\;\;\;\;\;;\;\;i=1,2,\ldots .S$$

where *S* is the number of output neurons, *R* is the dimension of the input vectors, *p_ij_* represents the *j* element of the input vector, and *w_ij_* symbolizes the *j* element of the *i* weight vector. The term BMU is defined, according to Lee & Scholz [15], as the neuron with the weight vector closest to the input variable *x*, as given by the equation:

(2) $$\left|x-m_{c}\right|=min(\left|x-m_{i}\right|)$$

where| |symbolizes the distance measure, *x* the input vector, *m* the weight vector, and *c* the subscription of the weight vector for the winning neuron. A more detailed description of the SOM algorithm can be found in the studies of Lee &Scholz [14], Aguilera et al. [40], Park et al. [43], and An et al. [44].

All the input parameters are transformed before presenting them to the SOM neural network, with the use of log transformation. The optimum map size of the SOM can be calculated based on the relationship:

(3) $$M\approx 5\sqrt{n}$$

where *n* is the data sample number and *M* is the number of neurons [44,45]. The SOM Toolbox for MatLab [46] was used for data simulations.

## 3. Results

### 3.1. PCA and Cluster Analysis Results

**Figure 3:**
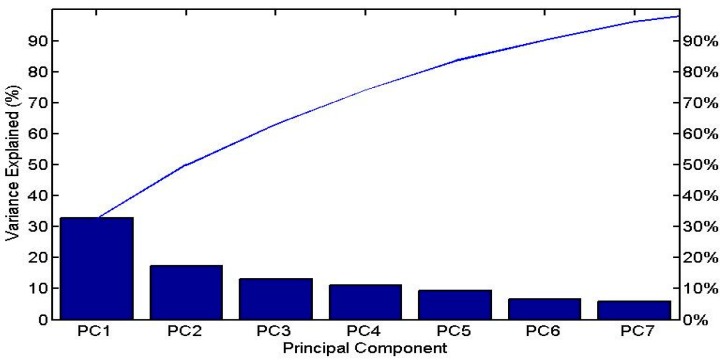
Pareto chart representing the percentage of variance explained by the principal components (PCs) of the PCA analysis.

The first four principal components (PCs) of the PCA analysis satisfy the criterion of the proportion of variance accounted for [31], and together they summarize 74.17% of the total variance. A Pareto chart is created, where the percentage of variance explained by each PC is presented (Figure 3). At the same time, the criterion of eigenvalue-one is met, as suggested by An et al. [44], by retaining the first four PCs (principal components).

A descriptive table for the first four PCs and their characteristics is created (Table 2).

**Figure 4:**
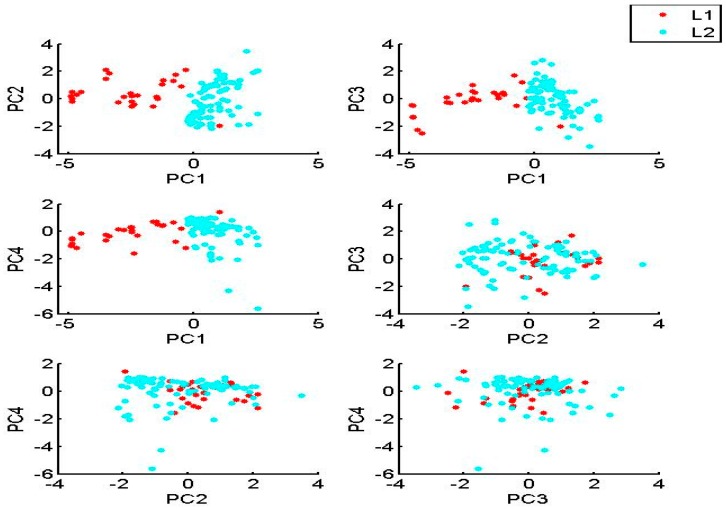
Bivariate plots between four different principal components (PCs) of Lake Megali Prespa (L1) and Lake Mikri Prespa (L2).

PC1 explains 32.68% of the total variance, and has strong negative loadings for SD and depth, and strong positive loadings for EC and phytoplankton Chl-a*.* PC1 seems to describe the relationships between SD, depth, EC and Chl-a*.* PC2 explains 17.29% of the total variance, and has strong positive loadings for pH, WT, and Chl-a*.* PC2 describes the relationship between the Chl-a and WT parameters. PC3 explains 13.08% of the total variance, and has a strong positive loading for DO and a strong negative loading for TP. PC4 explains 11.12% of the total variance, and has a strong negative loading for DIN. The bivariate plots for the PCs (Figure 4) reveal differences among the samples from Lake Megali Prespa, symbolized as L1 (n1 = 26), and the samples from Lake Mikri Prespa, symbolized as L2 (n2 = 89). The bivariate plots of PC1 versus the rest of the PCs show a clear separation between the samples from the two lakes.

**Figure 5:**
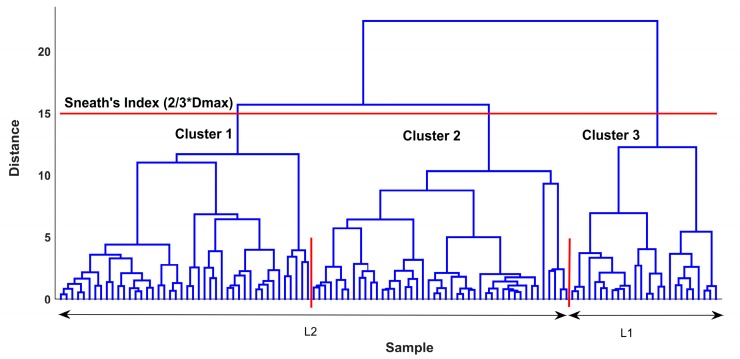
A dendrogram of similarity for the data samples from Lake Megali Prespa (L1) and Lake Mikri Prespa (L2). Based on Sneath’s criterion (red horizontal line), three clusters are formed (separated by the red vertical solid lines). *Dmax*: maximum distance.

The cluster analysis grouped the samples with respect to the water quality parameters. The cluster analysis divided the data into three clusters, where Ward’s linkage method and Euclidean distance measurements were used. The dendrogram constructed by the means of cluster analysis, shown in Figure 5, illustrates the arrangement of the clusters.

Cluster 3 corresponds to samples from Lake Megali Prespa, and cluster 1 and cluster 2 correspond to samples from Lake Mikri Prespa. A clear distinction is observed for the data from the two lakes, where all L1 samples are illustrated at the right side (branch) of the dendrogram and all L2 samples are illustrated at the left side (branch) of the dendrogram. The Sneath’s index of cluster significance calculated the cluster number with the use of a cutoff line for the dendrogram; the less restrictive criterion of Sneath’s index of cluster significance, equal to 2/3 of *Dmax*, was used.

### 3.2. SOM Algorithm Results

**Figure 6:**
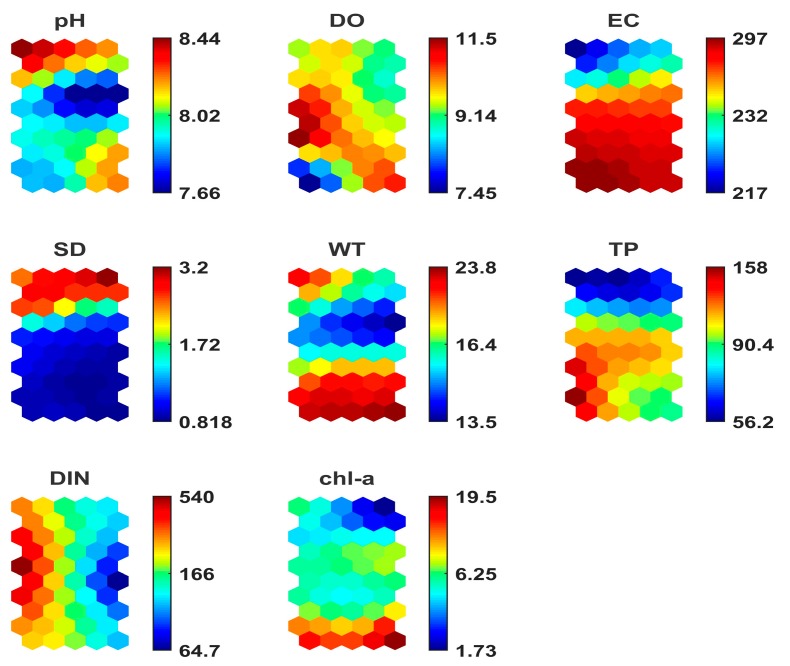
Visualization of the SOM’s component planes (CPs) for each environmental parameter, where the colorbars indicate the mapping of the data values.

A SOM with 10 × 5 neurons was created, based on the rule given by Equation (3). The components planes (CPs) are visualized in Figure 6. For the clustering of the SOM prototypes, the K-means algorithm method and the hierarchical algorithm method were used and evaluated in order to find the most appropriate clustering method for this modeling study.

**Figure 7:**
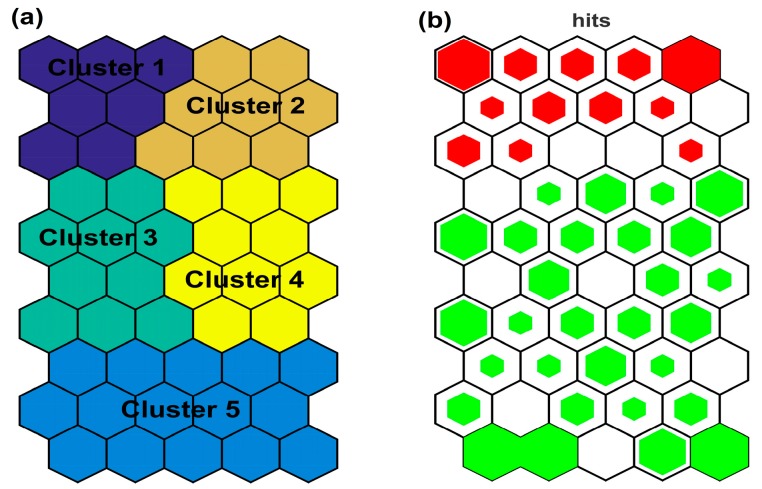
(**a**) Clustering of the SOM based on the K-means algorithm. (**b**) Hits histogram of SOM analysis representing the density of SOM hits. The green represents data from Lake Mikri Prespa, the red represents data from Lake Megali Prespa, and the empty nodes are associated with the absence of data samples.

The optimal number of clusters minimizes the Davies–Bouldin index when the SOM is implemented by the K-means algorithm [44,47]. In our case, the optimal cluster number was five (Figure 7). The L1 samples (Megali Prespa) correspond to cluster 1 and cluster 2, while the L2 samples (Mikri Prespa) correspond to cluster 3, cluster 4, and cluster 5. The SOM gave a clear classification of the samples from the two lakes.

The hits histogram distinguished the Megali Prespa data from the Mikri Prespa data. The difference between the data from Megali Prespa and data from Mikri Prespa is primarily associated with the water transparency (or SD). It is also reversely associated with the EC.

**Figure 8:**
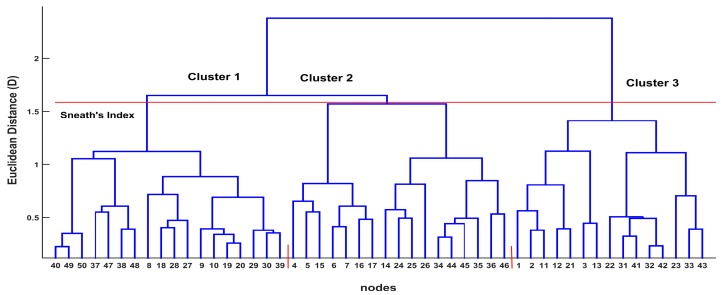
A dendrogram of similarity for the SOM’s prototype nodes. Based on Sneath’s less restrictive criterion (red horizontal line), three clusters are formed (separated by the red vertical solid lines).

The hierarchical cluster analysis performed on the SOM prototypes calculated three district groups for the investigated lakes. Ward’s linkage method and Euclidean distance measurement were used, and a dendrogram was derived (Figure 8). The existence of three clusters was computed with the use of the less restrictive significance of Sneath’s index (2/3 of *Dmax*). Based on the dendrogram, the L1 samples (Megali Prespa) are located on the right side (branch) and belong to cluster 3, while the L2 (Mikri Prespa) samples are located on the left side (branch) and belong to cluster 1 and cluster 2.

As expected, the sampling sites from the shallower Lake Mikri Prespa were associated with higher Chl-a values, as shallow lakes are more prone to nutrient-mixing eutrophication that might benefit algal production [48]. The TP values also increased as water depth decreased. It is documented that strong positive relationships exist between TP loadings and algal biomass [49]. No clear conclusions are extracted for the DO parameter. The pH parameter seems to have a negative relationship with the Chl-a, and generally the pH parameter is associated with eutrophication [24,50]. Besides the visualization of the results with the CPs, the SOM clusters can also provide useful information regarding the water quality parameter interactions. 

For the clustering of the SOM’s prototypes, two different clustering methods were used. The K-means algorithm method (see Table 3) and the hierarchical algorithm method (see Table 4) were applied. The mean values and standard deviation (SD) for each SOM cluster were calculated and are presented in Table 3 and Table 4. 

The K-means algorithm is considered a most suitable clustering method for the result implementation of this modeling study, as it calculated five clusters instead of the three that the hierarchical algorithm found. The existence of five clusters instead of three allows the more detailed examination of the environmental parameter interactions. For example, regarding the Lake Megali Prespa-associated clusters, the role of WT and therefore the seasonality effect is easily observed in Table 3, but no clear conclusions can be derived from Table 4.

## 4. Discussion

Unraveling and investigating trophic function mechanisms is a task of major importance, particularly for developing sustainable management and restoration plans for lakes with poor water quality. There is a large number of monitoring studies that have pointed out that nutrient enrichment has been the main cause of eutrophication, for example [50,51]. PCA and cluster analysis are statistical methods commonly used in such studies that often provide satisfactory results and useful conclusions about the main parameters associated with eutrophication processes. 

Conversely, SOMs models are considered to be more advanced modelling tools used in water quality modelling studies [47]. Modelling studies examining lake water quality by combining a SOM model and statistical methodologies are approaching more comprehensively the behaviour of the limnological parameters. Wang et al. [47] and An et al. [44] displayed valuable results with statistical methodologies, however the findings of the SOM model were much more practical and specific. The prevalence of the SOM method against PCA is related to the fact that the relationships among environmental variables are nonlinear, while the PCA is based on linear principles [52]. An important advantage of the SOM compared with the PCA method is based on its visualization abilities, provided by the SOM’s component planes. With the use of the component planes, the distribution of the component values is represented and direct visual examination is provided in order to allow correlations between several component planes that can be investigated simultaneously [53].

Another advantage of the SOM method compared with the classic statistical techniques is related to the dimension reduction provided by the SOM by projecting multidimensional data into a two-dimensional space. The dendrogram created by applying the hierarchical clustering method on the standardized raw data is usually overcrowded, making its implementation difficult. In contrast, the dendrogram resulting from the SOM prototypes is easily implemented. This is because the SOM neural network abridges the samples of the initial data set to a smaller set of prototypes, providing the possibility to construct a simplified dendrogram [54].

Representation by the CPs can be considered as an efficient and practical visualization tool for extracting useful conclusions regarding the trophic function of the two lakes. The L1 (Megali Prespa) data are correlated with high SD and low EC values, thus providing a clear separation from L2 (Mikri Prespa) data. The parameter SD seems to have a crucial role for clustering the data of the studied lakes. Meanwhile, the SD parameter has a negative correlation with the EC, since shallower lakes are often associated with higher EC. The high WT are linked with high Chl-a values, mainly for samples from Lake Mikri Prespa. Despite the rather small number of samples collected from the littoral zone of the Greek part of Lake Megali Prespa, the SOM managed to separate the data from the two lakes, and produced results comparable to those of the other statistical tools. The environmental parameters associated with algal production were visualized by the SOM component planes. Through the CPs, the visualization of all variables enables them to be examined simultaneously, and direct associations between the variables for specific value ranges of each examined variable can be obtained. In addition, PCA and cluster analysis do not always capture the hidden information that is provided by the data set. In contrast, the SOM can detect hidden patterns and recognize specific features of the data set which are often different in the short-term assessment [55]. The SOM revealed hidden patterns for each lake after careful examination of the CPs (Figure 6). Our results showed that the modelled environmental parameters, which are associated with algal production, followed a pattern influenced by the seasonality effect for each lake data set. Specifically, the K-means algorithm clustering results for Lake Mikri Prespa showed that the data of cluster 5 had higher values for the Chl-a and WT parameters than the other two clusters, suggesting the seasonality effect over Lake Mikri Prespa and the elevated algal production during hot months. The data that are associated with cluster 3 and cluster 4 had moderate values for Chl-a and WT, suggesting such conditions of these parameters during spring and autumn. However, no clear patterning is observed for the rest of the parameters. A deeper examination of the revealed patterns shows that for Lake Mikri Prespa, Chl-a presents its maximum value when the WT is also maximum; while at the same time, the TP presents its minimum and the DIN is relatively very low. Regarding Lake Megali Prespa, the minimum Chl-a value is associated with the maximum SD value, elevated WT value, and relative low TP and DIN values. In contrast, the maximum Chl-a value is associated with the maximums of the pH and WT parameter values, elevated DIN, and a very low TP value. These two clusters suggest an almost reversed patterning between them, where Lake Megali Prespa is affected by the seasonality to a lesser extent than the shallow Lake Mikri Prespa.

Additionally, the SOM more reliably visualizes nonlinear and heterogeneous data than the PCA method into a two-dimensional space [56]. The above observations highlight the SOM superiority compared with the PCA, since the SOM captures well the complex nonlinear mechanisms associated with algal production. However, the PCA did not manage to relate the parameter interactions well enough, limited by its linear nature as a method. For example, the nonlinear relationship between the Chl-a and TP parameters is well presented by the SOM through its CPs as was discussed for each lake data set, and specific information regarding the parameter interactions can be extracted based on the mapping of the data values. In contrast, the PCA (see Figure 4) provides only generalized information for a tendency regarding these parameter interactions. Because of this, the SOM is considered to be an innovating method compared with PCA and cluster analysis. Based on the SOM results, high nutrient concentrations were associated with elevated phytoplankton Chl-a values. High water temperature (WT) values were also correlated with the high Chl-a values. The role of water temperature as a key environmental parameter for controlling phytoplankton biomass in Lake Mikri Prespa was highlighted in a study by Tryfon & Moustaka-Gouni [57], where it was shown that an increase in biomass of cyanophytes occurred at temperatures greater than 16 °C. The SD depth was also found to be a critical parameter based on PCA, cluster analysis, and SOM. Both the cluster analysis method and the SOM method managed to distinguish between the samples from Lakes Mikri and Megali Prespa, mainly because of the SD depth variation. However, the SOM with the use of K-means algorithm distinguished the water quality parameters into five diverse clusters, providing a deeper examination of the parameter interactions, in contrast with the cluster analysis method, which gave only three clusters. The results presented in Table 3 showed that cluster 2 samples are characterized by lower Chl-a and lower WT values than cluster 1, reflecting the seasonal variations of algal productivity of Lake Megali Prespa. Additionally, cluster 3 and cluster 4, which are associated with Mikri Prespa, show noticeably different mean WT values compared with cluster 5. Cluster 5, in particular, seems to be associated with the hot months and the highest temperatures, where Chl-a has the highest mean value. On the other hand, cluster 3 and cluster 4 have lower WT and Chl-a mean values, probably representing conditions typical of the spring and autumn seasons.

The depth parameter, which is strongly correlated with SD depth, was not included as an input variable for the SOM model, because the aim of this modelling study was to focus on the mechanisms that are affecting algal production, and not the morphological characteristics of the lakes. In order to avoid bias in the results, it was decided that the depth parameter would not be included as an input parameter.

It is documented that low water transparency (SD depth) is associated with increased algal production [58,59], and Chl-a has an analogous relationship with the SD depth [60]. In a study by Zacharias et al. [61] that examined the biological and chemical characteristics of the major Greek lakes, it was stated that the SD depth is high in deep lakes and low in shallow lakes. The SOM model that managed to separate the two lakes matched the high SD depth with samples from Lake Megali Prespa and low SD depth values with samples from Lake MikriPrespa. As expected, the observation of the CPs associated the data samples of Lake Mikri Prespa with low SD depth values and increased Chl-a levels, and the data samples of Lake Megali Prespa with high SD depth values and low Chl-a levels.

The role of the SD depth has been given great attention in many studies, in order to discriminate possible environmental factors influencing the water quality between shallow and deep lakes. For example, in a study by Stefanidis et al. [62], two geomorphologically different lakes were examined, and it was found that the SD depth was a good indicator of the water quality deterioration and enhancement of eutrophication. The above monitoring examples, combined with the fact that the nutrient loading threshold for obtaining clear water conditions differs among climate zones [63], lead to the conclusion that modelling limnological data sets is a very case-sensitive task; while the mechanisms controlling limnological parameters are complex and their interactions are hard to examine. Therefore, the application of a SOM is ideal in studying water quality parameters and their interactions in lakes.

The increasing eutrophication that has been reported for the studied lakes [26] is mainly related to human activities. The last decades’ increased land use activities in the catchment’s area, such as agriculture, the encroachment of wetland areas, and the expansion of the irrigation system, have had a negative impact on the wetland biodiversity, enhancing the eutrophication processes [20]. This water quality deterioration was demonstrated by Loffler et al. [25], where a remarkable decrease of Secchi depth in Lake Megali Prespa, from 7.2 to 10 m in the 1950s to 3.2 m in the 1990s, was noted. The SOM managed to simulate the algal production process for the two lakes and identify the environmental parameter interactions. Therefore, SOM algorithms can be used as a tool that can aid management authorities in developing a guideline regarding the restoration measures that should be applied. Besides the water transparency that was one of the key factors associated with water quality, the water temperature was significantly correlated with phytoplankton Chl-a. Based on this finding, it would be interesting to further explore the effects of the anticipated rising of temperature, along with intensification of water level changes on the water quality and the processes of eutrophication. 

In our case, the SOM separated the two sampling areas and verified the good water quality status of Lake Megali Prespa. Regarding the shallow Lake Mikri Prespa, which is suffering the effects of eutrophication, the role of water transparency and water temperature should be considered when prioritizing management measures. Proposed management options should mitigate the excessive use of fertilizers, but should also consider other pressures, such as water abstraction, in order to reduce nutrient loadings and provide a relatively stable water level regime.

## 5. Conclusions

This modelling study presented different methodologies in order to examine the water quality of the investigated lakes. The results derived from the classic statistical methodologies were compared with the SOM neural network findings. The results from PCA, cluster analysis, and the SOM method agreed about the trophic function properties of the lakes, but the SOM results were more specific, and allowed direct associations between the water quality variables. The SOM neural network has a basic advantage derived from its visualization abilities. Based on our findings, the SOM neural network can be described as an innovating modelling tool that can be used autonomously or in parallel with the rest of the traditional modeling methods for successfully evaluating freshwater quality.

## Figures and Tables

**Table 1:** Statistical description of the seasonally measured environmental parameters for Lakes Megali Prespa and Mikri Prespa.

Variable	Mikri Prespa (*n* = 79)			Megali Prespa (*n* = 26)		
	Mean	Minimum	Maximum	Mean	Minimum	Maximum
pH	7.93	7.00	8.60	8.32	8	8.90
Dissolved oxygen (mg/L)	9.96	4.50	18.00	9.75	5.90	13.00
Electricalconductivity (μS/cm)	281.94	245.00	310.00	221.61	214	227.00
Secchi depth (m)	0.92	0.40	2.00	3.35	1.00	6.00
Water depth (m)	1.39	0.70	2.50	6.33	1.50	12.00
Water temperature (°C)	19.20	12.60	26.1	18.54	13.80	24.00
Total phosphorus (μg/L)	123.47	17.00	463.00	77.43	21.90	249.10
Dissolved inorganic nitrogen (mg/L)	319.07	28.40	2486.00	249.07	79.50	808.30
Chlorophyll-a *(*mg/m^3^)	10.76	1.10	42.70	4.01	0.40	14.50

**Table 2:** Principal components (PCs) percentage of variance, eigenvalues, and loadings for the examined environmental parameters.

Variable	PC1	PC2	PC3	PC4
pH	−0.1609	**0.6158**	0.1653	−0.0569
DO	−0.0350	−0.0237	**0.7513**	−0.1349
EC	**0.4908**	−0.0198	−0.0993	−0.0736
SD	**−0.5300**	0.0511	−0.2700	−0.1709
Depth	**−0.5171**	0.1026	−0.2527	−0.1701
WT	0.2051	**0.6144**	−0.1386	0.0279
TP	0.1613	−0.2545	**−0.4452**	0.1072
DIN	0.1248	−0.1500	−0.0275	**−0.9302**
Chl-a	**0.3230**	**0.3766**	−0.2085	−0.1936
Eigenvalue	2.94	1.55	1.18	1.01
Variance explained (%)	32.68	17.29	13.08	11.12
Cumulative variance (%)	32.68	49.97	63.05	74.17

DO: dissolved oxygen; EC: electrical conductivity; SD: Secchi disk depth; WT: surface water temperature; TP: total phosphorus; DIN: dissolved inorganic nitrogen; Chl-a: chlorophyll-a. The significant correlation coefficients are shown with bold characters.

**Table 3:** Statistical description of the SOM’s clusters found using the K-means algorithm method, based on mean values and standard deviation (SD).

Variable	Cluster 1		Cluster 2		Cluster 3		Cluster 4		Cluster 5	
	Mean	SD	Mean	SD	Mean	SD	Mean	SD	Mean	SD
pH	8.4	0.22	8.18	0.15	7.91	0.35	7.76	0.33	8.06	0.39
DO	10.19	2.05	9	1.34	11.54	2.74	9.82	2.65	9.55	1.99
EC	220.06	3.65	229.45	19.89	276.13	15.31	265.14	16.95	290.21	12.24
SD	3.23	1.78	3.33	1.78	0.98	0.34	0.96	0.28	0.85	0.33
WT	20.38	3.6	16.25	1.73	15.73	0.35	15.54	3.74	23.36	2.19
TP	77.38	41.46	73.61	57.9	145.5	2.74	112.89	43.98	122.6	80.34
DIN	331.76	232.62	135.12	36.23	481.37	15.31	101.75	36.62	195.54	83.21
Chl-a	5.64	3.7	1.9	0.72	6.37	0.34	7.22	3.36	15.4	9.74

**Table 4:** Statistical description of the SOM’s clusters found using the hierarchical algorithm method, based on mean values and standard deviation (SD).

Variable	Cluster 1		Cluster 2		Cluster 3	
	Mean	SD	Mean	SD	Mean	SD
pH	8.07	0.37	7.77	0.33	8.30	0.22
DO	9.62	2.37	10.78	2.54	9.66	1.84
EC	289	11.89	267.69	17.17	224.24	14.04
SD	0.89	0.38	0.94	0.22	3.27	1.75
WT	22.82	2.66	14.68	2.16	18.30	3.55
TP	126.64	81.25	124.46	55.43	75.71	48.45
DIN	183.44	82.82	316.05	326.88	244.36	199.01
Chl-a	13.41	9.96	7.35	3.2	3.98	3.34
